# Retraction

**DOI:** 10.1002/cam4.5232

**Published:** 2022-09-08

Retraction: ‘Effects of miR‐145‐5p through NRAS on the cell proliferation, apoptosis, migration, and invasion in melanoma by inhibiting MAPK and PI3K/AKT pathways,’ Sha Liu, Guozhen Gao, Dexiong Yan, Xiangjun Chen, Xingwei Yao, Shuzhong Guo, Guirong Li, Yu Zhao, *Cancer Medicine*. 2017; 819–833: The above article, published online on March 23, 2017 in Wiley Online Library (https://onlinelibrary.wiley.com/doi/10.1002/cam4.1030) has been retracted by agreement between the authors, the journal's Editor‐in‐Chief, Dr. Stephen Tait, and John Wiley & Sons Ltd.

The retraction has been agreed following allegations raised by a third party. Multiple flaws and inconsistencies between the results presented and the experimental methods described were found, as well as instances of image manipulation across several figures. As a result, the editors consider the conclusions of this article to be invalid.

